# Chronic obstructive pulmonary disease: a disease of old age?

**DOI:** 10.7189/jogh.08.020306

**Published:** 2018-12

**Authors:** Esther A Boudewijns, Giridhara R Babu, Sundeep Salvi, Aziz Sheikh, Onno CP van Schayck

**Affiliations:** 1 Care and Public Health Research Institute, Maastricht University, Maastricht, the Netherlands; 2 Public Health Foundation of India, Indian Institute of Public Health-Hyderabad, Bangalore campus, Bangalore, India; 3 Chest Research Foundation, Pune, India; 4 Centre of Medical Informatics, Usher Institute of Population Health Sciences and Informatics, The University of Edinburgh, Edinburgh, UK

**Figure:**
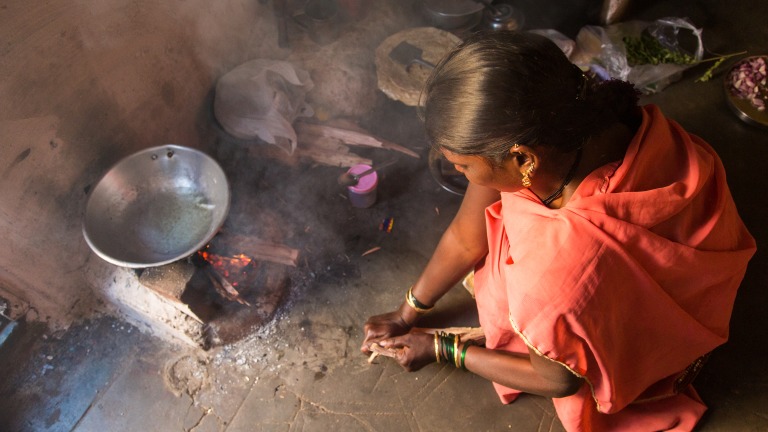
Photo: A young woman with increased risk of COPD due to indoor air pollution. From the collection of Martijn van der Sanden (tijnmedia), used with permission

Chronic obstructive pulmonary disease (COPD) is one of the leading causes of morbidity and mortality globally, with more than 174 million people suffering from this disease. Historically, COPD has been considered as a disease affecting the elderly, with a preponderance in male smokers. Over the last few years, this is disputed by several studies indicating a growing health burden of COPD among women [1] and non-smokers [2]. Our historical view of COPD as a disease of elderly male smokers is thus slowly changing towards an all-encompassing range of susceptibilities for COPD. However, what is overlooked is that COPD is not a disease exclusively found among elderly people, but that it is often prevalent among younger people as well.

According to Jenkins [1], there are major health differences between women and men in COPD risk, progression and outcomes, which poses challenges for its management. Women are more likely to be underdiagnosed, smoke less, have a lower body mass index (BMI), are often exposed to other polluting sources such as biomass fuel and are more susceptible to developing COPD. Besides the growing recognition of the burden among women, several studies have demonstrated that risk factors other than smoking are equally or even more closely associated with COPD [2,3]. Emerging evidence suggests that approximately 25-45% of patients with COPD in low- and middle-income countries (LMICs) have never smoked [2,4]. For example, approximately 56% persons affected with COPD in urban slums and 85% in rural areas in India never smoked [4,5]. Exposure to biomass fuels is probably the leading cause of COPD in younger women [2,3]. Despite limited efforts to characterise risk profiles in vulnerable communities, available evidence indicates that risk factors unrelated to smoking contribute significantly to the development of COPD, especially in LMICs [2].

The available data regarding the prevalence of COPD in different age categories is scarce. A study in rural Uganda demonstrated that COPD was most prevalent among women under the age of 40 years [6]. The prevalence decreased with age, which may be explained by the healthy survivor effect. This suggests that the elderly group most likely consisted of healthy elderly people, and the unhealthy elderly had passed away. Furthermore, de Marco (2004) showed a relatively high prevalence of COPD in young adults aged 20-44 years in the European Community Respiratory Health Survey [7]. It seems that COPD is often underdiagnosed in younger patients, which suggests that it might be even more prevalent than assumed. Better orientation regarding diagnosing COPD in younger people is urgently needed in order to prevent underdiagnosis of the disease.

A history of smoking is one of the major risk factors for COPD and exposure to it is often analysed using pack-years, defined as the product of the number of packages smoked per day and the number of years a person has smoked [8]. Several studies have demonstrated a direct association between pack-years and the incidence and prevalence of COPD, with higher risks associated with an increasing number of pack-years and a direct association between an increasing number of cigarettes per day and lung function decline. High numbers of pack-years are usually the result of either lengthy exposure or a high smoke intensity. Exposure to smoke from biomass fuels can cause a higher intensity compared to tobacco smoke, with particulate matter (PM_2.5_) levels reaching 1500 µg/m^3^ [9], which is 60 times higher than the levels recommended by the World Health Organization’s (WHO) guidelines. It, therefore, seems evident that there is a direct association between cumulative exposure to biomass smoke, as well as the combined risk with smoking, and the incidence of COPD, leading to COPD in people younger than 40 years [10]. Moreover, looking at the prevalence rates among certain groups, this conclusion seems to be confirmed. Several studies suggest that women are more likely to present with COPD before the age of 60 than men [1]. Furthermore, the burden among women seems to be higher in LMICs than in high-income countries (HICs) [1]. COPD due to risk factors unrelated to smoking seems to be more apparent in LMICs than in HICs [2]. High exposure to indoor air pollution from the use of biomass fuels seems to be the most probable factor resulting in the high burden of young women affected with COPD in LMICs, combined with second-hand smoke exposure and high levels of atmospheric pollution [2]. Biomass smoke contributes to approximately 50% of all deaths from COPD in LMICs [2]. In these countries, justified stress on the effect of smoking has often overshadowed the equal or greater impact of addressing other risk factors unrelated to smoking, such as biomass fuels. To tackle the enormous burden of COPD in these LMICs, in addition to smoking cessation, interventions should focus on reducing indoor air pollution, such as improvement of traditional stoves for people who have no access to cleaner fuels due to financial or logistic constraints [11].

We thus argue that the main focus when diagnosing COPD should not be on age, as this will lead to underdiagnosis and an underestimation of the problem, specifically in LMICs. It is important to consider contextually specific interventions to reduce indoor air pollution to mitigate the COPD burden in young persons.
